# Conceptualizing sexual and gender-based violence in European asylum reception centers

**DOI:** 10.1186/s13690-019-0351-3

**Published:** 2019-05-31

**Authors:** Charlotte Oliveira, Maria do Rosário Oliveira Martins, Sónia Dias, Ines Keygnaert

**Affiliations:** 1 Global Health and Tropical Medicine, GHTM, Instituto de Higiene e Medicina Tropical, IHMT, Universidade NOVA de Lisboa, Rua da Junqueira 100, 1349-008 Lisbon, Portugal; 2 Centro de Investigação em Saúde Pública, Escola Nacional de Saúde Pública, Universidade NOVA de Lisboa, Lisbon, Portugal; 3 International Centre for Reproductive Health, Department of Public Health and Primary Care, Faculty of Medicine & Health Sciences, Ghent University, Ghent, Belgium

**Keywords:** Sexual and gender based violence, Sexual violence, Refugees, Asylum-seekers, Migrants, Asylum reception centres, Professionals, Conceptualization, Prevention

## Abstract

**Background:**

Sexual and gender based violence (SGBV) is a major public health problem and a violation of human rights. Refugees, asylum seekers and migrants are exposed to a constant risk for both victimization and perpetration. Yet, in the context of European asylum reception centers (EARF) professionals are also considered to be at risk. Our study explores the conceptualization of SGBV that residents and professionals have in this specific context. Further, we intent to identify key socio-demographic characteristics that are associated with SGBV conceptualization for both groups.

**Methods:**

We developed a cross-sectional study using the Senperforto project database. Semi-structured interviews were conducted with residents (*n* = 398) and professionals (*n* = 202) at EARF. A principal component analysis (PCA) was conducted to variables related with knowledge on SGBV. *Chi-square test* and *Fisher’s exact test* were applied to understand if significant statistical association exists with socio-demographic characteristics (significant level 0.5%).

**Results:**

The majority of residents were male (64.6%), aged from 19 to 29 years (41.4%) and single (66.8%); for professionals the majority were women (56.2%), aged from 30 to 39 years (42.3%) and married (56.8%). PCA for residents resulted in 14 dimensions of SGBV representing 83.56% of the total variance of the data, while for professionals it resulted in 17 dimensions that represent 86.92% of the total variance of the data. For both groups differences in SGBV conceptualization were found according to host country, sex, age and marital status. Specific for residents we found differences according to the time of arrival to Europe/host country and type of accommodation, while for professionals differences were found according to legal status and education skills.

**Conclusion:**

Residents and professionals described different conceptualization of SGBV, with specific types of SGBV not being recognized as a violent act. Primary preventive strategies in EARF should focus on reducing SGBV conceptualization discrepancies, taking into account socio-demographic characteristics.

## Background

Sexual and Gender-based Violence (SGBV) is a major public health problem and a violation of human rights [1, 2]. SGBV encompasses gender-stereotyped acts of violence, based on unequal power relations and denying human dignity, rights and development [1, 3].

Considering the global challenge of (forced) migration [4], United Nations High Commissioner for Refugees (UNHCR) defines SGBV as “(…) violence that is directed against a person on the basis of gender or sex. It includes acts that inflict physical, mental or sexual harm or suffering, threats of such acts, coercion and other deprivations of liberty (….)” [1]. SGBV comprises five categories, namely, physical, psychological, sexual, socio-economic violence and harmful cultural practices [1]. SGBV conceptualization is a matter of judgement, affected by cultural beliefs, social norms and values [5]. What is considered a violent behavior varies according to specific determinants such as socio-cultural and historical conditions [6].

Referring to SGBV conceptualization within the context of European Union (EU) policy documents, SGBV in general, and more specifically in migrants, has been framed as violence against women [7, 8]. Yet, literature has demonstrated that female, male and transgender refugees, asylum seekers (AS) and migrants are vulnerable to SGBV [4, 9–13]. In a study on SGBV among refugees, AS and undocumented migrants in European asylum reception centers (EARF) a high percentage of multiple types of SGBV was reported in all sexes [14]. A study done in Belgium and the Netherlands found a high prevalence of direct or indirect SGBV exposure among migrant: 87/223 respondents had been personally victimized since their arrival in Europe. The majority of perpetrators were male (74.0%), and 69.3% of victims were female (male victims were 28.6%). Also, asylum related professionals were found to be assailants in one fifth of the reported violence [13].

A socio-ecological approach is described in the literature as an understanding model for SGBV [1, 2, 15]. The model assumes SGBV as the result of a permanent and dynamic interaction between health determinants at four levels: individual, relational, community and society [1, 2, 15]. A combination of these levels triggers the patterns of SGBV [16, 17]. At individual level, research has shown that women and girls, especially the impoverished are more prone to victimization [13, 18]. Recent evidence demonstrates that boys and men are also exposed to sexual violence [19]. In the context of EARF, both females and males have a tendency to be victims and/or perpetrators [14]. Furthermore, age [20], attained education and cultural beliefs appears to be important determinants when addressing SGBV [10]. At a relational level, children exposed to a violent context are more susceptible of becoming victims and/or perpetrators [21]. Further, a systematic review highlights that immigrant adolescents are exposed to high rates of violence [22]. From a community and societal perspective, studies have shown that an important determinant for sexual violence among refugees, AS and undocumented migrants is their restricted legal status [7] and the migration process itself [3].

In the context of migration, it becomes relevant to engage with affected communities [23] and to understand legal power relations triggered by society constructed knowledge, beliefs and norms that undermine refugees, AS and migrants, threatening their human rights and putting them at higher risk of SGBV [24]. Primary prevention of SGBV should focus on measures ensuring ‘basic condition for sustainable and effective change’ [25]. In this sense, a wide conceptualization of SGBV from an individual, relational, community and societal perspective is needed to promote a comprehensive prevention approach to violence [26]. Moreover, the intersectional nature of SGBV should be acknowledged while addressing preventive measure [8, 27].

Our study aims to expand the understanding of SGBV conceptualization, in a vulnerable population of refugees, AS and migrants on the one hand and in professionals working with these communities in EARF on the other. Further, we identify socio-demographic characteristics of both groups that can be associated with SGBV conceptualization.

## Methods

### Study design

A cross-sectional study was conducted using data from the Senperforto Project developed in eight European countries (Belgium, Greece, Hungary, Ireland, Malta, The Netherland, Portugal and Spain). The main objective of Senperforto was to explore what knowledge, attitude, practice (KAP), and needs of professionals and residents from EARF were, in order to develop a gender-balanced European Frame of Reference for both beneficiaries [28]. Senperforto applied a community based participatory research methodology, mobilizing stakeholders – AS and refugees, asylum reception professionals, policy makers, civil society (…) – from the participating countries in the community advisory boards. Further, community researchers – professionals and/or residents that showed good social and communication skills – were trained (standardized training course) to conduct semi-structured interviews. Finally, a KAP survey was conducted.

For a detailed description of Senperforto project and methodology we refer to the article Sexual and Gender-based violence in the European asylum and reception sector: a perpetuum mobile [14].

### Participants, sample and data collection

The Senperforto Project participant sample included 600 residents and professionals living and working in EARF. Residents refer to refugees, AS, and undocumented migrants. Professionals refer to services and health care providers working in the facilities. The inclusion criteria for the residents (*n* = 398) implied being member of the most numerous groups of asylum seeking and unaccompanied minor communities in the host country of research. They had to be staying at, or just having left, an asylum reception facility in the country of research. For professionals (*n* = 202), they had to work, or just stopped working at asylum reception facilities. Regarding the selection of facilities (open or closed (detention) facilities, reception or return centres, private accommodation, urban/rural, unaccompanied minors facilities, AS centres and refugee centres) all official reception facilities were listed; and facilities were selected in order to have at least one category of facility represented among the respondents. If more than one centre was available for a certain type of facility, centres were chosen randomly.

Also, a geographical distribution over the country of research was conducted and taken into account to the feasibility of the study. Considering that the situation of the asylum reception sector in each partner country differs, the sampling strategy was adapted to the local situation. In all countries random sampling was used except for Spain and the Netherlands where convenient sampling was applied due to political constraints [14].

.Data was obtained through semi-structured interviews that were conducted by well-trained community researchers. The questionnaire included data on socio-demographic characteristics of the participants and continued with three dimensions of research [1]: knowledge of the respondent on types of SGBV, on occurrence of violence and existence of prevention measures [2]; attitudes regarding SGBV and its prevention within EARF [3]; and a part on their evaluation of effectiveness of existing SGBV prevention and response measures and suggestions. Our study focuses on the first part of the questionnaire, which consisted of 82 closed questions coded with a Likert scale (I fully agree, I agree, Neutral, I do not agree, I fully disagree). Questions described the different acts of SGBV as put forward in the UNHCR guidelines on SGBV prevention and response [1] and inquired on a gender conceptualization, i.e. did they perceive the described behaviour as a violent act when it was done to girls and women, and subsequently if the same act happened to boys and men? Finally, the questionnaire was translated and back translated into the languages of the main groups of AS in the 8 participating countries, as well as the official language of each participating country (Arabic, Dari, Dutch, English, French, Greek, Hungarian, Portuguese, Romanes, Somali, Spanish, Russian, Maltese, Amharic and Tigrigna). A pilot test was done with members of the community advisory board. Prior to the interview respondents had agreed with the community researcher on the chosen language and sex of the interviewer. The interviews were conducted one-to-one at a private place in or near the asylum reception facility.

The Senperforto project applied the ethical and safety guidelines in researching violence recommended by WHO and UNHCR. Furthermore, it complied with the local ethical requirements and received ethical approval from Ghent University Hospital Ethical Committee [B67020096667].

### Statistical methods

The questionnaires from Senperforto project included quantitative and qualitative data. For qualitative data, a framework analysis technique was used, further categorization and introduction into IBM® SPSS software. Quantitative data was introduced directly in IBM® SPSS software database. For our study we used a factor analysis approach using Principal Component Analysis (PCA) [29] for a factor extraction and Varimax rotation, to reduce the volume of the data. We conducted a multivariate analysis of 82 variables regarding SGBV knowledge. PCA analyses data representing observations described by dependent but inter-correlated variables. The goal is to extract the most important information from the original data and to convert this new information as a set of new variables, i.e. principal components (PC) [29]. These PC’s were analyzed and named dimensions of SGBV, according to the questions with higher loading result from PCA output. The next step consisted of the recodification of the PC’s – dimensions of SGBV – into nominal variables, each of them with three categories (negative, neutral and positive) according to the crosscut values for lower and upper barrier outliers. The lower fence outliers matched with the group of people that fully agreed with the dimension of violence in analysis while the upper fence outliers matched with the ones that fully disagreed.

Subsequently, we selected specific socio-demographic characteristics for residents and professionals. Commonly analyzed socio-demographic characteristics included: country of research (from here called host country), sex, age, marital status, religion, status according to immigration law and type of facility living/working (detention center, open reception center, local reception initiative, return center). Specifically for residents, we included the variables: having children, year of arrival to Europe and to hosting country, kind of accommodation (house, apartment, container, room, homeless…), attained education, daily activity in the country of origin and hosting country. For professionals we included: number of languages speaking and number of languages actually needed at work (here interpreted as language skills), to be working in a reception center by the time of questionnaires and the current occupation. Statistical tests were applied as the Chi-square Test and Fisher’s exact test, to understand if significant statistical association exist at the 5% significance level.

## Results

### Profile of respondents

The majority of residents were male (64.6%), aged 19–29 years old (41.4%) and single (66.8%); for professionals the majority were women (56.2%), aged 30–39 years old (42.3%) and married (56.8%). For residents, we had 53 different countries of origin, the more representatives were Somalia (20.9%), Afghanistan (11.1%), Nigeria (8.5%), Guinea Conakry (6.3%) and Iraq (4.5%). Regarding educational level, 48.5% of residents had the secondary level of education, 25.6% had primary education, 14.1% university degree and 10.8% no education. For professionals occupational background 50.0% were social assistants, 21.0% security or administration related, 19.8% directors (20%), and 9.0% health related professionals. Table 1 presents an overview of socio-demographic characteristics for both groups.

**Table 1:** Socio-demographic characteristics of residents and professionals

	Residents		Professionals		Total	
	N 398	%	N202	%	N 600	%
Host country						
Belgium	61	15.3	32	15.8	93	15.5
Greece	36	9.0	30	14.9	66	11.0
Hungary	68	17.1	21	10.4	89	14.8
Ireland	63	15.8	32	15.8	95	15.8
Malta	61	15.3	30	14.9	91	15.2
Netherlands	33	8.3	5	2.5	38	6.3
Portugal	53	13.3	37	18.3	90	15.0
Spain	23	5.8	15	7.4	38	6.3
Marital Status						
Single	266	66.8	65	32.7	331	55.4
Engaged	6	1.5	4	2.0	10	1.7
Married/Legally cohabiting	99	24.9	113	56.8	212	35.5
Prior relation. Not anymore	27	6.8	17	8.5	44	7.4
Missing	0	–	3	–	3	–
Religion						
Yes	374	94.2	130	65.0	504	84.4
No	23	5.8	70	35.0	93	15.6
Missing	1	–	2	–	3	–
Year of arrival in Europe						
< 2000	8	2.0	15	50.0	23	5.4
2000–2004	36	9.1	9	30.0	45	10.5
2005–2008	193	48.6	6	20.0	199	46.6
2009–2010	160	40.3	0	0.0	160	37.5
Missing	1	–	172	–	173	–
Year of arrival to host country						
< 2000	3	0.8	15	46.9	18	4.2
2000–2004	29	7.3	9	28.1	38	8.9
2005–2008	186	47.0	8	25.0	194	45.3
2009–2010	178	44.9	0	0.0	178	41.6
Missing	2	–	170	–	172	–
Legal Status						
Asylum Seeker	246	62.3	0	0.0	246	47.3
National Citizen	0	0.0	109	87.2	109	21
Temporary Residence Status	83	21.0	5	4.0	88	16.9
Recognized Refugee	38	9.6	8	6.4	46	8.8
Refused Asylum Seeker	16	4.1	0	0.0	16	3.1
Undocumented	9	2.3	0	0.0	9	1.7
Immigrant worker	0	0.0	3	2.4	3	0.6
Other	3	0.8	0	0.0	3	0.6
Missing	3	–	77	–	80	–

### SGBV conceptualization

#### Residents

When analyzing the results of the multivariate analysis of principal components, we found 14 new variables, representing 83.56% of the total variance of the data. These new variables were analyzed according to the questions with higher PCA output loading, labeled as dimensions of SGBV according to UNHCR definition [1] and represents residents SGBV conceptualization. The questions that correspond to each dimension are described in Table 2.

**Table 2:** Principal component analysis for residents: representative questions and output loading (Varimax variation)

Residents	
SEXUAL VIOLENCE	PCA Loading output
PC12 – Sexual innuendo	
Unwelcome and unwanted sexual comments or invitations to girls/women.	0.862
And if this happens to boys/men?	0.876
PC4 – Visual sexual Harassment	
Made to watch photos of naked persons as a girl/woman?	0.820
And if this happens to boys/men?	0.817
Made to watch porn as a girl/woman?	0.802
And if this happens to boys/men?	0.767
PC9 – Marital Rape	
Unwanted sex within a relationship and/or marriage to a girl/woman?	0.754
And if this happens to boys/men?	0.793
PC1 – Abuse, rape and trafficking	
Unwelcome penetration of the vagina and/or anus by an organ or by an object of girl/woman.	0.831
And if this happens to boys/men?	0.831
Forced prostitution of girls/women?	0.817
And if this happens to boys/men?	0.819
Sexual slavery/trafficking of girls/women?	0.749
And if this happens to boys/men?	0.802
Rape of girls/women as a weapon of war?	0.791
And if this happens to boys/men?	0.789
PSYCHOLOGICAL VIOLENCE	
PC3 – Humiliation	
Unwelcome remarks and comments from nonsexual nature to girls/women.	0.767
And if this happens to boys/men?	0.787
Teasing, showing no respect, racist or discriminating comments to a girl/woman?	0.716
And if this happens to boys/men?	0.728
PC6 – Confinement	
Someone denying a girl/woman to be together with their partner in private?	0.751
And if this happens to boys/men?	0.700
HARMFUL CULTURAL PRACTICES	
PC10 – Denial of education of girls and women	
Neglecting female children, denial from education to female children?	0.623
And if this happens to boys/men?	0.653
PC14 – Genital mutilation	
Circumcision of girl/woman?	0.450
PC11 – Early marriage	
Child marriage of a girl/woman?	0.803
And if this happens to boys/men?	0.817
PC5 - Honor killing and Maiming	
Killing a girl/woman in the name of family honor?	0.751
And if this happens to boys/men?	0.745
SOCIO-ECONOMIC VIOLENCE	
PC13 – Discrimination	
Being treated differently by other people because of being a girl/woman?	0.569
And if this happens to boys/men?	0.603
PC2 - Denial of opportunities and services	
Denial of access to education, health assistance or remunerated employment because of the residence status of a girl/woman.	0.792
And if this happens to boys/men?	0.790
Denial of access to education, health assistance or remunerated employment because of being a girl/woman.	0.774
And if this happens to boys/men?	0.763
PC8 – Denial of access to exercise civil, social, economic rights	
As a girl/woman to be isolated, confined and/or deprived of liberty of movement	0.644
And if this happens to boys/men?	0.637
PC7 – Social exclusion/ostracism based on sexual orientation	
Being treated differently by other people because of the sexual orientation of girl/woman.	0.853
And if this happens to boys/men?	0.855

#### Professionals

The multivariate analyze for the group of professionals resulted in 17 new variables representing 86.92% of the total variance of collected data. These new variables were analyzed and labelled dimensions of SGBV [1] representing professionals SGBV conceptualization. The representative questions of each dimension of SGBV are described in Table 3.

**Table 3:** Principal component analysis for professionals: representative questions and output loading (Varimax variation)

Professionals	
SEXUAL VIOLENCE	PCA Loading output
PC15 – Sexual innuendo	
Unwelcome and unwanted sexual comments or invitations to girls/women.	0.619
And if this happens to boys/men?	0.600
PC3 – Visual sexual harassment	
Made to watch somebody undress as a girl/woman?	0.858
And if this happens to boys/men?	0.879
Made to watch photos of naked persons as a girl/woman?	0.887
And if this happens to boys/men?	0.817
PC14 – Denudement	
Having to undress in front of other people watching as a girl/woman?	0.698
And if this happens to boys/men?	0.799
PC1 –Abuse, Rape and Trafficking	
Unwelcome penetration of the vagina and/or anus by an organ or by an object of girls/women?	0.930
And if this happens to boys/men?	0.930
Trafficking of people for their organs?	0.833
And if this happens to boys/men?	0.833
PC10 – Sexual exploitation	
Sex with a girl/woman in exchange for survival, food for the children, shelter, money, papers, other favors.	0.916
And if this happens to boys/men?	0.916
PHYSICAL VIOLENCE	
PC8 – Physical assault without permanent consequences	
Physical assault with no permanent consequences (e.g. hitting, kicking, pulling)	0.773
And if this happens to boys/men?	0.790
PC5 – Physical assault with permanent consequences	
Physical assault with permanent consequences (e.g. burning, stabbing, maiming)	0.877
And if this happens to boys/men?	0.877
Killing a girl/woman in the name of family honor?	0.798
And if this happens to boys/men?	0.843
PSYCHOLOGICAL VIOLENCE	
PC 12 – Threat and humiliation	
Threatening of girls/women with unwelcome not sexual acts (make you feel scared.…)	0.548
And if this happens to boys/men?	0.549
Teasing, showing no respect, racist or discriminating comments to a girl/woman?	0.546
And if this happens to boys/men?	0.546
PC9 – Verbal violence	
Unwelcome remarks and comments from nonsexual nature to girls/women.	0.759
And if this happens to boys/men?	0.759
PC13 – Confinement, individual level	
As a girl/woman to be isolated, confined and/or deprived of liberty of movement	0.720
And if this happens to boys/men?	0.642
PC6 – Relational violence	
Someone denying a girl/woman to be together with his or her partner in private?	0.863
And if this happens to boys/men?	0.862
Someone denying a girl/woman to be together with her parents or children in private.	0.812
And if this happens to boys/men?	0.812
PC16 – Parental relational violence	
Someone denying a girl/woman to fulfill her role as a mother (no money for food)	0.669
And if this happens to boys/men?	0.669
HARMFUL CULTURAL PRACTICES	
PC17 – Genital mutilation	
Circumcision of girl/woman?	0.417
And if this happens to boys/men?	0.632
PC7 – Early marriage	
Child marriage of a girl/woman?	0.882
And if this happens to boys/men?	0.862
PC 11 – Honor killing and maiming	
Injuring a girl/woman in the name of family honor?	0.853
And if this happens to boys/men?	0.853
SOCIO-ECONOMIC VIOLENCE	
PC2 – Denial of opportunities and services	
Denial of access to education, health assistance or remunerated employment because of the ethnic background of a girl/woman	0.874
And if this happens to boys/men?	0.874
Denial of access to education, health assistance or remunerated employment because of the residence status of a girl/woman.	0.846
And if this happens to boys/men?	0.846
PC4 – Social exclusion/ostracism	
Being treated differently by other people because of the sexual orientation of a girl/woman?	0.794
And if this happens to boys/men?	0.806
Being treated differently by other people because of the ethnic background of a girl/woman?	0.780
And if this happens to boys/men?	0.780

Table 4 shows the conceptualization of SGBV for residents and professionals from EARF grouped according to UNHCR SGBV definition [1].

**Table 4:** Residents and professionals – SGBV conceptualization, grouped according to UNHCR SGBV definition

Residents	Professionals
Sexual Violence	
Sexual innuendo	Sexual innuendo
Visual sexual harassment	Visual sexual harassment
–	Denudement
Marital rape	–
Abuse, rape and trafficking	Abuse, rape and trafficking
–	Sexual exploitation
Physical Violence	
–	Physical assault without permanent consequences
–	Physical assault with permanent consequences
Psychological Violence	
Humiliation	Threat and humiliation
Confinement	Verbal violence
–	Confinement – individual level
–	Relational violence
–	Parental relational violence
Harmful Cultural Practices	
Denial of education of girls and women	–
Genital mutilation	Genital mutilation
Early marriage	Early marriage
Honor killing and Maiming	Honor killing and Maiming
Socio-economic Violence	
Discrimination	–
Denial of opportunities and services	Denial of opportunities and services
Denial of access to exercise civil, social, economic rights	–
Social exclusion/ostracism based on sexual orientation	Social exclusion/ostracism

The association between each dimension of SGBV conceptualization and resident’s socio-demographic characteristics or professionals’ characteristics are presented in Tables 5 and 6, respectively. Our results describe whether, or not, what is considered a specific behavior/sexual act as violence is different according to sex, age, kind of accommodation (…). We will now describe the significant results first for residents and subsequently for professionals.

**Table 5:** Residents – SGBV conceptualization and socio-demographic characteristics (*p*-values: Chi-square Test and Fisher’s exact test)

Socio-demographic characteristics of Residents	Dimensions of SGBV Concept													
	Sexual				Psychological		Harmful Cultural Practices				Socio-economic			
	PC 12	PC 4	PC 9	PC 1	PC 3	PC 6	PC 10	PC 14	PC 11	PC 5	PC 13	PC 2	PC 8	PC 7
Host country	**0.010**	0.167	0.127	**0.001**	0.266	0.183	0.155	0.571	0.482	**0.001**	0.678	0.842	0.078	0.086
Sex	0.268	0.650	0.580	0.056	0.829	1.000	0.886	0.897	0.070	**0.004**	0.374	1.000	0.852	0.305
Age	0.185	0.625	**0.001**	0.212	0.806	**0.032**	0.059	0.545	0.470	**0.042**	0.616	1.000	0.105	0.174
Marital status	0.842	0.273	0.754	0.281	0.362	0.204	**0.033**	0.363	0.189	0.580	0.253	0.565	0.911	0.716
Having Children	0.104	1.000	0.243	0.288	0.289	0.125	0.502	0.530	0.295	0.874	0.498	1.000	1.000	0.801
Religion	1.000	1.000	1.000	0.344	0.344	0.065	**0.019**	0.520	0.425	0.489	0.489	1.000	1.000	0.275
Status immigration Law	0.195	0.087	1.000	0.321	0.626	0.798	1.000	0.124	0.328	1.000	0.161	1.000	0.458	0.064
Year of arrival Europe	0.708	1.000	0.544	0.281	1.000	0.773	0.679	0.484	0.603	0.679	0.079	1.000	0.340	**0.018**
Year of arrival to host country	0.458	1.000	0.513	0.075	1.000	0.737	0.618	0.420	0.543	1.000	0.295	1.000	0.280	**0.007**
Type of reception facility living	0.394	0.646	0.792	0.062	1.000	0.332	1.000	1.000	1.000	0.471	0.436	1.000	1.000	1.000
Kind of accommodation	**0.026**	0.439	**0.001**	0.388	0.934	0.056	0.951	0.520	1.000	0.123	0.728	0.645	0.207	0.603
Attained education	**0.016**	0.668	0.647	0.415	0.899	0.560	0.447	**0.033**	0.558	0.180	0.074	0.611	0.940	0.390
Daily activity country of origin	0.587	0.215	1.000	0.232	0.065	1.000	0.453	0.902	0.308	0.412	0.288	1.000	0.659	**0.046**
Daily activity host country	**0.037**	1.000	0.233	0.412	0.502	0.650	0.834	0.467	0.176	0.278	0.758	0.308	0.070	0.744

Significant p-value *p* < 0.05 bolded

PC 12: Sexual innuendo; PC 4: Visual sexual harassment; PC 9: Marital rape; PC 1: Abuse, rape and trafficking; PC 3: Humiliation; PC 6: Confinement; PC 10: Denial of education of girls and women; PC 14: Genital mutilation; PC 11: Early marriage; PC 5: Honor killing and maiming; PC 13: Discrimination; PC 2: Denial of opportunities and services; PC 8: Denial of access to exercise civil, social and economic rights; PC 7: Social exclusion/ostracism based on sexual orientation

**Table 6:** Professionals – SGBV conceptualization and socio-demographic characteristics (*p*-values: Chi-square Test and Fisher’s exact test)

Socio-demographic characteristics	Dimensions of SGBV Concept																
	Sexual violence					Physical Violence		Psychological violence					Harmful Cultural Practices			Socio-economic	
	PC 15	PC 3	PC 14	PC 1	PC 10	PC 8	PC 5	PC 12	PC 9	PC 13	PC 6	PC 16	PC 17	PC 7	PC 11	PC 2	PC 4
Host country	0.363	0.142	**0.030**	0.516	**0.002**	**0.015**	0.687	0.388	0.180	**0.004**	0.391	0.556	0.594	**0.001**	0.725	0.081	0.473
Sex	0.451	0.072	0.736	0.078	0.360	0.256	0.503	0.509	0.333	0.932	1.000	0.502	**0.043**	0.884	0.345	**0.049**	0.498
Age	0.618	1.000	0.106	**0.021**	0.647	0.443	0.937	0.126	0.068	0.801	1.000	0.488	0.618	0.871	1.000	0.441	0.483
Marital status	0.753	0.133	0.734	0.451	**0.014**	0.469	0.512	0.381	**0.042**	0.053	0.533	0.773	0.609	0.189	0.489	0.616	0.500
Religion	0.328	1.000	0.847	0.072	0.862	0.588	0.247	0.822	0.210	1.000	0.609	1.000	0.135	0.213	0.816	0.456	0.498
Status immigration Law	0.146	**0.037**	0.680	0.125	0.784	0.440	1.000	0.234	0.450	**0.001**	1.000	0.433	0.851	0.607	0.783	0.639	0.301
Type of reception Center working	0.851	0.551	0.397	0.497	0.073	0.487	0.829	0.189	0.833	0.282	0.630	0.763	0.734	**0.027**	0.373	0.229	0.300
Number of languages speaking	0.624	0.617	0.381	0.969	0.782	0.267	0.541	0.651	0.587	0.660	0.185	0.233	0.197	0.308	0.997	0.440	1.000
Number of languages needed at work	**0.012**	**0.038**	**0.000**	0.131	0.031	0.377	0.470	0.434	0.387	**0.040**	0.078	0.970	0.706	**0.047**	0.454	0.615	0.519
Actually working in a Reception center	0.192	1.000	0.125	0.425	0.817	0.649	0.762	0.064	1.000	**0.005**	1.000	0.674	0.192	**0.031**	0.443	0.568	0.685
Current occupation	0.720	0.593	0.063	0.696	0.213	0.193	0.747	0.235	0.079	0.460	0.833	0.469	0.836	0.353	0.528	0.930	0.819

Significant p-value p < 0.05 bolded

PC 15: Sexual innuendo; PC 3: Visual sexual harassment; PC 14: Denudement; PC 1: abuse, rape and trafficking; PC 10: Sexual exploitation; PC 8: Physical assault without permanent consequences; PC 5: Physical assault with permanent consequences;; PC 12: Threat and humiliation; PC 9: Verbal violence; PC 13: Confinement, individual level; PC 6: Relational violence; PC 16: Parental relational violence; PC 17: Genital mutilation; PC 7: Early marriage; PC 11: Honor killing and maiming; PC 2: Denial of opportunities and services; PC 4: Social exclusion and ostracism

### Residents

#### Sexual violence

For residents, sexual innuendo conceptualization was associated with the host country (*p* = 0.010), kind of accommodation (*p* = 0.026), the level of education of residents (*p* = 0.016) and daily activity in the host country (*p* = 0.037). This mean that residents living in Belgium and Ireland, in a container, studio or room, with an education (primary, secondary or higher), or do not have a job in the host country tend to disagree that sexual innuendo is a type of SGBV.

Marital rape was associated with the age of residents (*p* = 0.001), and the kind of accommodation where they were living in (p = 0.001). Youth and adults’ residents (0–39 years old) or residents living in containers, room or studio tend to disagree that marital rape is a form of violence. Abuse, rape and trafficking was associated with host country (p = 0.001). Residents that tend to disagree were hosted in Portugal and Spain.

#### Psychological violence

The concept of confinement was significantly associated with age (*p* = 0.032), meaning that residents aged until 18 years old tend to disagree with confinement as a form of violence.

#### Harmful cultural practices

Denial of education for girls was associated with marital status (*p* = 0.033) and the fact of having (or not) a religion (*p* = 0.019). Single residents tend to fully agree with this as a form of violence. The conceptualization of genital mutilation as a form of violence was associated with attained education (p = 0.033).

Honor killing and maiming conceptualization was associated with the country of research (*p* = 0.001), sex (male or female) (*p* = 0.004) and age (*p* = 0.042) of residents. Residents hosted in Belgium and Greece, male or aged from 19 to 39 years old tend to disagree with this concept as a form of violence.

#### Socio-economic violence

The concept of social exclusion based on sexual orientation was associated with the time of arrival to Europe or hosting country (*p* = 0.018 and 0.007), and daily activity in the country of origin (*p* = 0.046). Residents that arrived recently to the host country or Europe (less than 5 years) or used to have a job in the country of origin tends to fully disagree that social exclusion based on sexual orientation is a form of violence.

### Professionals

#### Sexual violence

For professionals, sexual innuendo was associated with language skills (*p* = 0.012). Professionals with good language skills (at least 2 EU languages) tend to fully disagree. Visual sexual harassment conceptualization was associated with language skills (*p* = 0.038) and status immigration law (*p* = 0.037). The tendency to disagree was found in professionals without the national citizenship and basic language skills (1 EU language). Denudement was associated with the hosting country and language skills (*p* = 0.030, *p* = 0.000, respectively). Professionals from Portugal or with basic language skills (1 EU language) tend to fully disagree. Abuse, rape and trafficking conceptualization was different according to the age of professionals (*p* = 0.021). Older professionals (> then 40 years old) tend to fully disagree. Further, sexual exploitation as a form of violence was associated with hosting country, marital status and language skills (*p* = 0.002, *p* = 0.014 and *p* = 0.031). Tendency to fully disagree were found in professionals from Malta, Netherlands and Portugal, married or with good language skills (1 EU and 1 non-EU language).

#### Physical violence

The concept of physical assault without permanent consequences as form of violence was significantly associated with hosting country (*p* = 0.015). Professionals working in Hungary tend to fully disagree.

#### Psychological violence

Verbal violence was associated with marital status (*p* = 0.042), with single professionals disagreeing more than the average of respondents. Confinement (individual level) as a form of violence was associated with host country (*p* = 0.004), status of immigration (*p* = 0.001), language skills (*p* = 0.040) and the fact of being working (*p* = 0.005). Professionals that have a tendency to fully disagree were from Belgium and the Netherland, without the national citizenship, with good language skills (2 EU languages) or with a current job at the time of the questionnaire.

#### Harmful cultural practices

Genital mutilation was associated with professionals’ sex (*p* = 0.043), meaning that male professionals tend to fully disagree with it as an act of violence. Early marriage as form of violence was different according to the hosting country, type of reception facility, language skills or the fact of being working (*p* = 0.001, *p* = 0.027, *p* = 0.047 and *p* = 0.031). Professionals working in Belgium, in open reception facilities or with good language skills tend to fully disagree.

#### Socio-economic violence

Denial of opportunities and services as a form of SGBV was associated with sex (*p* = 0.049) and female professionals tend to fully disagree that it represents a form of violence.

In sum, our results suggest that professionals from EARF considered more behaviors as violence than residents.

## Discussion

The scientific understanding of violence and more specifically SGBV is primordial [6] to enhance primary preventive measures. In this sense, if we want to prevent violence in the EARF, understanding the knowledge that residents and professionals have regarding SGBV conceptualization is needed. Our study explored SGBV conceptualization according to residents and professionals from EARF, covering a myriad of countries of origin of the refugees, AS and migrants. Our results show a disparity between what is, or what is not considered a violent behavior. Professionals have shown to have a wider knowledge then residents, considering more acts as violence. We believe this can be related to residents – refugees, AS and undocumented migrants – being described as more vulnerable to SGBV and professionals assuming a privileged position and control towards residents [24].

Conceptualization is a process of development and clarification of concepts; it shapes the field in which a concept is understood, measured and evaluated [30]. Different SGBV conceptualization can be found in the literature. Walby [8] refers that different definitions are used for assault and for rape, which are inconsistent and out of alignment with international law. Also, different SGBV conceptualizations were found in our results for residents and professionals. To consider that definitions of violence have evolved through multiple variations according to the field and the range of forms of violence [30]. A consistent and coherent measurement of violence against women and men will benefit accuracy while measuring changes in society and effectiveness of public services [8]. Given this, we believe a common SGBV conceptualization should be considered while addressing preventive measures. The requisite for developing information, education and communication (IEC) interventions addressing SGBV has already been acknowledged by UNHCR (2003). We believe our results call for the urgent need for IEC interventions, addressing what is, or what is not an SGBV act.

For both groups differences in SGBV conceptualization were found based on specific socio-demographic characteristics. As for gender, our results evoke no differences in SGBV conceptualization. Moreover, the fact that a violent act is directed to a girl/woman or a boy/man is equally considered violence, even though the majority of victims continue to be women [13] However, moving from SGBV conceptualization to specific types of SGBV differences arise. When conducting association tests between types of SGBV and the gender of our respondents we found significant associations. A more in-depth analysis suggests male residents tend to disagree that honor killing and maiming is an SGBV act when compared with the mean average of our respondents. Moreover, male professionals disagree with genital mutilation as a form of SGBV, and female professionals tend to disagree with the denial of opportunities as a form of SGBV.

Another relevant association was found between age and specific forms of SGBV. Results from our study, found that professionals aged above 40 tended to disagree that “abuse, rape and trafficking” is a form of SGBV. This association is particularly screaming for action, once we assist to professionals working with persons, already in a vulnerable situation, and assuming that a behavior legally punishable by law is acceptable. Considering that professionals play an important role in SGBV prevention, and the fact that they are in a privileged position to mitigate SGBV, we believe that our results are screaming for action. From one side we assist to professionals having a broader SGBV conceptualization when compared with residents. However, professionals aged above 40, do not consider abuse, rape and trafficking as a form of SGBV. In this sense, we believe there is a need for a strict screening when engaging professionals to work in EARF and continuous sensitization and training on SGBV. Our results are aligned with previous evidence reporting the requirement for healthcare workers’ regular training [25], integrated and widespread preventive and response measures [14]. Furthermore, professionals and persons in power working with migrants and refugees have been identified as potential perpetrators of SGBV [12, 24, 25]. In EARF context, professionals have been identified as potential perpetrators of SGBV, especially socio-economic violence [14].

Specific types of SGBV not being recognized as a violent act is of major importance while addressing preventive measures in asylum centers. Residents and professionals must have a complete and equal knowledge regarding all types of SGBV to avoid being victims and/or aggressors. Placing SGBV in a public health perspective, we can assume SGBV conceptualization is the baseline for primary prevention [5]. Furthermore, significant association with socio-demographic characteristics have arisen from our results. This fact shows the importance of recognizing the intersectionality of SGBV concepts [8, 27] with characteristics, such as gender, age, social status. We call for an urgent action from all stakeholders to increase the knowledge on SGBV of residents and professionals, based on IEC interventions, as the baseline to prevent violence before it occurs.

Future pertinent research regards the potential association between SGBV conceptualization and case disclosure. Moreover, it is of utmost importance to have a clear and in-depth understanding of professionals’ SGBV conceptualization. The fact that professionals might perpetuate SGBV acts, and exercise a higher power relation towards residents, represents a call for intervention. We challenge researchers to go beyond the understanding of professionals’ SGBV conceptualization and to consider the influence of it with the potential perpetuation of violence. Another relevant aspect to consider in the future regards the evaluation of primary preventive measures, and specifically the focus on promoting and implementing a widespread SGBV conceptualization among residents, professionals and host population. If we reach a level where professionals and residents have similar SGBV conceptualization, will we still witness such high levels of SGBV?

Even though relevant findings were described it is important to acknowledge potential limitations. The Senperforto project applied multi-types of sampling methods, as random and representative sampling were not possible in all countries. However, even though our results cannot be generalized, we believe it can be transferable to similar populations in comparable contexts, in a sense that a broad SGBV conceptualization is presented in our research – understanding refugees, AS and undocumented migrants’ perspective and also professional’s perspective. Specifically related with SGBV conceptualization, we cannot exclude that community researchers conducting the interviews during the implementation of Senperforto project, could have had a different SGBV conceptualization, even with the implementation of a standardized training.

Stepping out of EARF, it would be pertinent to compare SGBV conceptualization between migrants and hosting population, once public health policies should be adapted to the cultural and structural context. Moreover, it is important to consider the challenge of having refugees, AS and undocumented migrants with different SGBV conceptualization “integrated” in European countries, especially if they have a narrow concept. Accordingly, we believe migrants might be exposed to higher vulnerability to both victimization and perpetration. Considering the recent migration wave to European countries, it urges to address this issue. SGBV conceptualization needs to be addressed equally, not only for migrants and professionals, but also for hosting populations. What is or what is not an SGBV act should not differ according to a migration status. By not doing it, we believe European countries and its representatives might be increasing migrants’ vulnerability and inducing obstacles to their integration.

## Conclusion

Residents and professionals from European asylum centers have a different concept of what SGBV entails with professionals considering more acts as violence then residents. However, types of SGBV were considered equally violent if afflicted upon female or male. Some acts that were not considered violence by the professionals are legally a crime, increasing the perpetration risk.

The Socio-Ecological Model as an explanatory model of SGBV helps moving from the individual conceptualization of SGBV to a societal conceptualization considering the influences of relational, community and societal factors [31].

SGBV conceptualization is the core to primary prevention of SGBV and it should focus on harmonizing the concept, IEC activities, training and “collegiate” discussion/participatory activities towards consensus and European policies. What is considered (or not) a violent behavior should be taken into consideration if we want to mitigate SGBV.

We call for the development, implementation and monitoring of European-wide SGBV prevention programs in EARC context, aligned with SGBV conceptualization of the target population.

## Abbreviations

EARF: European asylum reception facilities
SGBV: sexual and gender-based violence
AS: asylum-seeker
UNHCR: United Nations High Commissioner for Refugees
EU: European Union
PCA: principal component analysis
PC: principal component
KAP: knowledge, attitudes and practices
IEC: information, education and communication
